# Acute Necrotizing Pancreatitis With Early Pseudocyst Formation Following Uncomplicated Laparoscopic Cholecystectomy: A Rare Postoperative Complication

**DOI:** 10.7759/cureus.94714

**Published:** 2025-10-16

**Authors:** Grace Lee, Nicholas Villar, Joseph Vo, Zakaria Abd Elmageed

**Affiliations:** 1 Biochemistry, Edward Via College of Osteopathic Medicine, Monroe, USA; 2 Orthopaedics, Edward Via College of Osteopathic Medicine, Monroe, USA; 3 Emergency Medicine, Edward Via College of Osteopathic Medicine, Monroe, USA; 4 Pharmacology, Edward Via College of Osteopathic Medicine, Monroe, USA

**Keywords:** general internal medicine, pancreas disease, pancreatic pseudocyst (ppc), pancreatic stent, surgery general

## Abstract

We present the case of a 60-year-old woman who was admitted with acute pancreatitis, cholelithiasis, and cholecystitis, and subsequently underwent an uneventful laparoscopic cholecystectomy. Two weeks postoperatively, she returned to the hospital with severe epigastric pain radiating to the back, accompanied by nausea and vomiting. Contrast-enhanced computed tomography (CT) imaging revealed acute necrotizing pancreatitis with early pancreatic pseudocyst formation at the junction of the pancreatic body and tail, without evidence of biliary obstruction or retained stones. She was managed with intravenous fluids, broad-spectrum antibiotics, bowel rest, analgesia, and underwent endoscopic retrograde cholangiopancreatography (ERCP) with pancreatic duct stent placement to reduce ductal pressure. She improved clinically and was discharged after one week of hospitalization, but returned two weeks later with signs of sepsis. Pancreatic pseudocysts are rare complications of pancreatitis, more often associated with chronic cases, and typically develop several weeks after inflammation or ductal injury. Their occurrence following an uncomplicated cholecystectomy in the absence of biliary obstruction or retained stones is exceptionally uncommon. This case underscores the diagnostic and management challenges of post-cholecystectomy pancreatitis complicated by pseudocyst formation and highlights the importance of interval imaging, multidisciplinary evaluation, and conservative management in stable patients. Awareness of this rare complication is essential to prevent unnecessary interventions while ensuring timely escalation of care when indicated.

## Introduction

Pancreatic pseudocysts are a rare complication of pancreatitis with an incidence of 0.5 to 1 per 100,000 adults per year [1]. It was first described in 1966 as an “intramural gastric pseudocyst” made of amylase, containing fluid occurring after chronic pancreatitis [2]. Pancreatic pseudocysts can develop following both acute and chronic pancreatitis, with a higher prevalence in men and greater incidence in chronic cases [3]. Since 1966, pancreatic pseudocysts have been found to occur around 4-6 weeks following an episode of pancreatitis or by direct trauma to the pancreatic ducts by stones, strictures or protein plugs. Due to the inflammation or trauma, fluid containing pancreatic enzymes extravasates into the parenchyma and forms a “pseudocyst” surrounded by a non-epithelialized wall of fibrous and granulation tissue, unlike true cysts, which are lined by epithelium [1]. Pancreatic pseudocysts are commonly found in the lesser sac; however, they have been found to involve other locations, including the retroperitoneum, inguinal canal, pleura, and even the mediastinum [4].

In most cases, pancreatic pseudocysts are self-limiting and resolve during the treatment of pancreatitis [5]. Patients who are symptomatic experience epigastric pain that may be referred to the left hypochondrium or radiate to the back, along with other symptoms, including early satiety, nausea or vomiting, fevers, or, less commonly, jaundice. Their symptoms may be exacerbated by eating food, which may cause marked weight loss in some patients [6]. If there is diaphragmatic involvement, pain may be pleuritic and radiate toward the shoulder [7]. Physical examination may reveal abdominal tenderness or an occasional palpable abdominal mass.

Diagnosis of a pseudocyst cannot be made until 4 weeks after the onset of pancreatitis. If the diagnosis is suspected, contrast-enhanced computed tomography (CT) and/or magnetic resonance imaging (MRI) are used to confirm the diagnosis. Other options for imaging include transabdominal ultrasound, endoscopic retrograde cholangiopancreatography (ERCP), and endoscopic ultrasound (EUS) [6]. In the past, surgical removal was the mainstay of treatment; however, it has been found that not all pancreatic pseudocysts require intervention. The American College of Gastroenterology (ACG) and the International Association of Pancreatology (IAP)/American Pancreatic Association (APA) guidelines recommend supportive treatment for asymptomatic cases [8]. Interventions like endoscopic and percutaneous drainage are reserved for symptomatic patients or those who have complications, including but not limited to suppuration, perforation, and rupture into the abdominal cavity or hollow organs [6,9].

We present a patient who developed a pancreatic pseudocyst following an uncomplicated laparoscopic cholecystectomy. This case is being reported due to its rare and complex presentation and etiology. Acute pancreatitis post-cholecystectomy is rarely reported, especially in cases where pseudocysts develop, retained common bile duct stones, or biliary obstructions.

## Case presentation

A 60-year-old female presents to the emergency department (ED) for extreme right upper quadrant abdominal pain triggered by greasy foods, associated with nausea, vomiting, and intermittent diarrhea. She has no past medical history outside of chronic pain managed with cyclobenzaprine, diclofenac, and gabapentin. A diagnosis of acute pancreatitis, cholelithiasis, and cholecystitis was made and the patient underwent an uneventful laparoscopic cholecystectomy. The patient then completed 2 weeks of meropenem (1 g IV every 8 hours) and was then discharged. The patient has given written and verbal consent for reporting this case.

Two days after discharge, the patient presented to the ED with a sudden onset of severe epigastric pain radiating to the back, accompanied by persistent nausea and multiple episodes of non-bloody vomiting. There were no fevers, chills, or recent alcohol use. On examination, the patient was hemodynamically stable but appeared in significant discomfort. Abdominal examination revealed marked tenderness in the epigastrium without rebound or guarding.

Admission laboratory evaluation showed an elevated serum lipase level of 293 U/L (reference range: 0-160 U/L), C-reactive protein of 10.4 mg/dL (reference level: <0.3 mg/dL), and an erythrocyte sedimentation rate of 49 mm/h. The complete blood count demonstrated mild leukocytosis with a white blood cell count of 12.3 ×10³/µL and thrombocytosis. Liver function tests and triglycerides were within normal limits (Table 1).

**Table 1:** Admission laboratory findings (Necrotizing pancreatitis presentation)

Test	Lab Result	Reference Range
Lipase	293 U/L	0-160 U/L
C-reactive protein	10.4 mg/dL	<0.3 mg/dL
Erythrocyte sedimentation rate	49 mm/h	≤ 30 mm/h
WBCs	12.3 × 10³/µL	4.0-10.5 × 10³/µL
Platelets	426 × 10³/µL	150-400 × 10³/µL
ALT	26 U/L	7-45 U/L
AST	27 U/L	8-43 U/L
Triglycerides	148 mg/dL	<150 mg/dL

**Figure 1:**
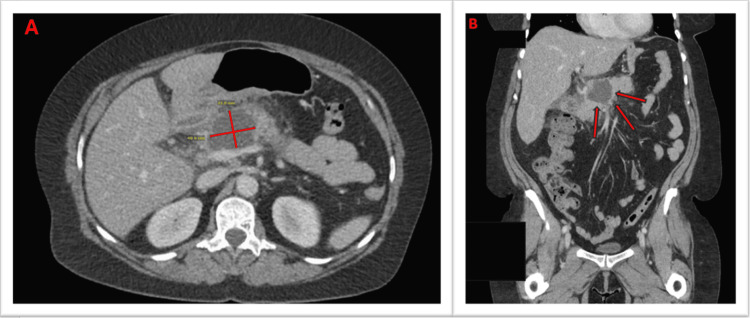
Axial and coronal view contrast-enhanced CT scan of the abdomen Contrast-enhanced CT scan of the abdomen depicts mesenteric stranding with a small amount of ascites. The pancreas showed a 5.1 cm × 4.1 cm hypodensity focus of the proximal aspect of the pancreas. The distal pancreas is unremarkable. Diffuse peripancreatic stranding. No dilatation of the pancreatic duct. Also noted in the peritoneum was a relatively new, irregular, lobulated, and loculated fluid collection of the right upper quadrant near the gallbladder fossa and caudate lobe. Panel A depicts an axial view, while panel B depicts a coronal view.

An initial IV contrast-enhanced CT scan of the abdomen revealed findings consistent with acute necrotizing pancreatitis involving the junction of the pancreatic body and tail (Figure 1). There was an associated peripancreatic fluid collection suggestive of an early pancreatic pseudocyst and evidence of secondary duodenitis with inflammatory stranding along the descending portion of the duodenum. No retained common bile duct stones or biliary obstruction was observed.

The patient was admitted with a diagnosis of acute necrotizing pancreatitis. Management included intravenous hydration, broad-spectrum antibiotics (meropenem 1 g IV every 8 hours and piperacillin-tazobactam 4.5 g IV every 6-8 hours), bowel rest, antiemetics, and hydromorphone (Dilaudid). General surgery was consulted, and no emergent intervention was recommended, given the immaturity of the pseudocyst and absence of infected necrosis.

**Figure 2:**
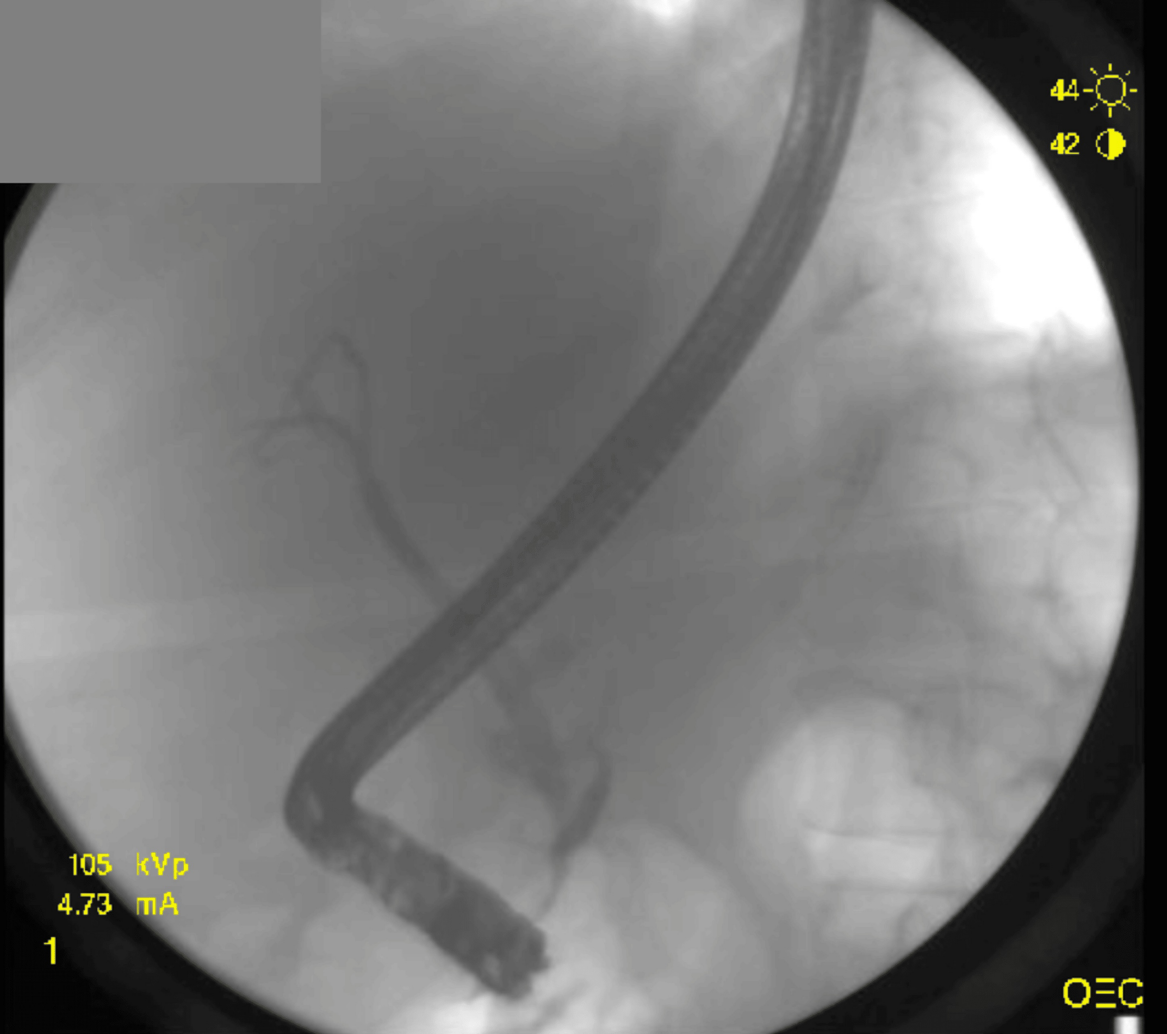
ERCP ERCP depicts normal opacification of the common bile duct and intrahepatic ducts, without visible stones, strictures, or dilatation. It suggests a normal-appearing biliary tree on this fluoroscopic frame. ERCP: endoscopic retrograde cholangiopancreatography

After about one week of supportive care, the patient underwent ERCP with placement of a pancreatic duct stent to facilitate drainage and reduce ductal pressure (Figure 2). The procedure was uneventful. Over the following days, the patient experienced progressive clinical improvement, with resolution of pain and normalization of leukocytosis. The patient was discharged after approximately one week of hospitalization with instructions for close outpatient follow-up.

Two weeks after discharge, the patient returned to the ED with recurrent severe abdominal pain. On presentation, vital signs were within normal limits. Laboratory analysis demonstrated a white blood cell count of 16 ×10³/µL, up from 8 ×10³/µL at the time of discharge, along with an elevated serum lipase level, while liver enzyme levels remained within normal limits (Table 2).

**Table 2:** Additional laboratory findings (Pancreatic pseudocyst presentation)

Test	Result	Reference Ranges
WBC	16 ×10³/µL	4.0-10.5 ×10³/µL
Platelets	540 ×10³/µL	150-400 ×10³/µL
Lipase	261 U/L	0-160 U/L
ALT	15 U/L	7-45 U/L
AST	20 U/L	8-43 U/L
CRP	181.7 mg/dL	<0.3 mg/dL

**Figure 3:**
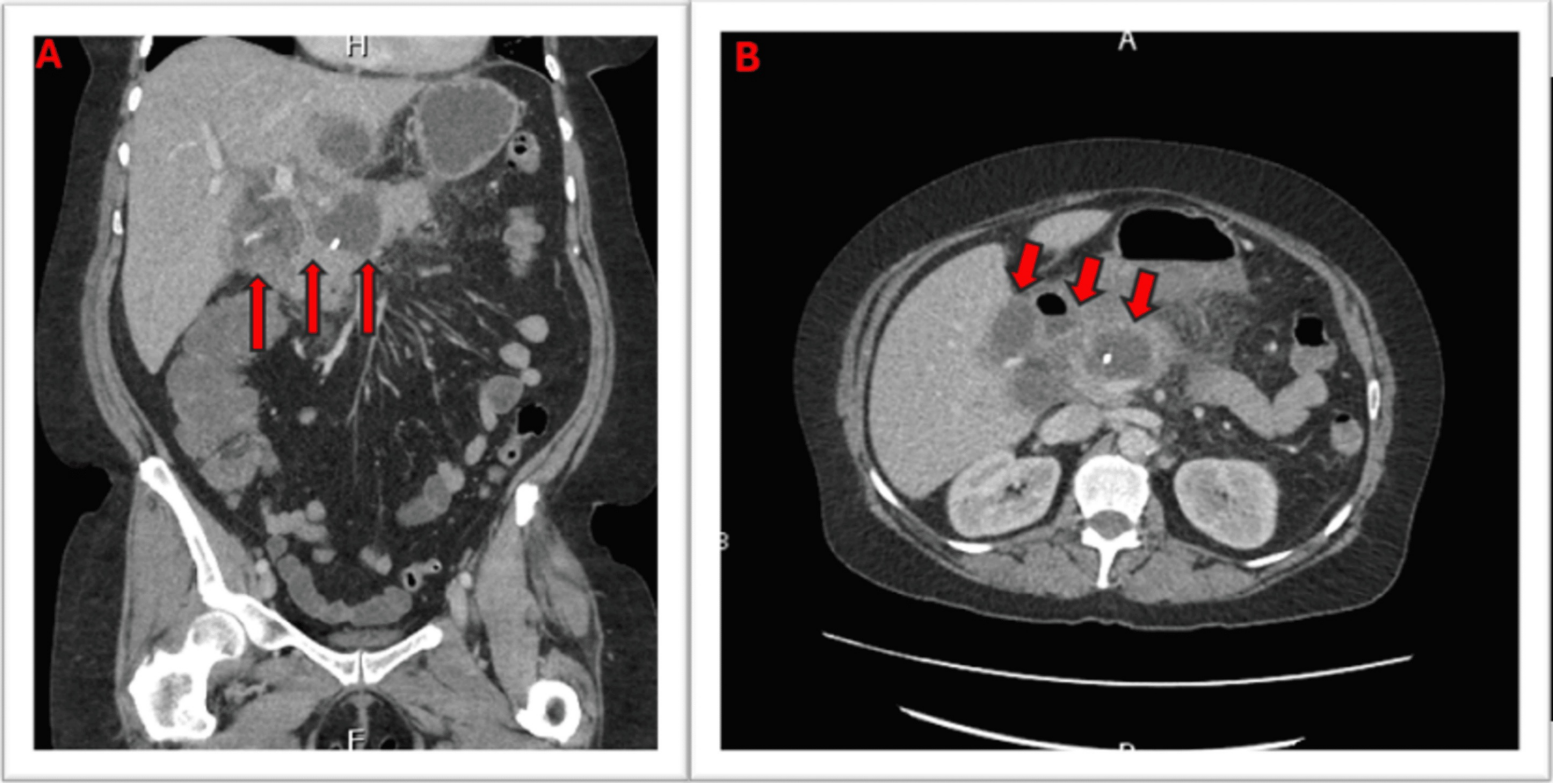
Contrast-enhanced CT abdomen and pelvis IV contrast-enhanced CT abdomen and pelvis: Interval placement of pancreatic stent. A slight decrease in the size of the fluid collection of the pancreatic neck. Upper abdominal/peripancreatic stranding and loculated fluid collection of the right upper quadrant are relatively stable. A small amount of intrapelvic free fluid has been resolved. Panel A depicts a coronal view, while panel B depicts an axial view.

A repeat contrast-enhanced CT scan of the abdomen and pelvis showed a slight reduction in the size of the previously noted pseudocyst, with persistent inflammatory changes and peripancreatic stranding. A loculated fluid collection in the right upper quadrant remained relatively stable compared to prior imaging (Figure 3).

The patient was admitted for evaluation and management of a possible infected fluid collection or developing sepsis and was restarted on intravenous piperacillin-tazobactam (3.375 g every 6 hours). Surgical and gastroenterology consultations recommended continued conservative management, as there were no clear indications for immediate drainage, and the fluid collections remained relatively stable on imaging.

## Discussion

Pancreatic pseudocysts are uncommon complications of pancreatitis that typically spontaneously resolve. The incidence is higher in males, which may follow the trend of pancreatitis, which is also more prevalent among men. Pseudocysts are slightly more common in cases of chronic pancreatitis, with incidence rates between 20-40%, versus acute pancreatitis, with incidence rates between 5-16%. This may be explained by the prolonged course of inflammation and progressive fibrosis characteristic of chronic pancreatitis [1,10]. However, pseudocyst formation as a direct complication of laparoscopic cholecystectomy (LC) is even more uncommon, with very few cases mentioned in the literature; a more recent case from 2022 demonstrates a pseudocyst formation 8 days following LC for acute gallstone pancreatitis [11].

Although pseudocysts are more commonly seen in chronic pancreatitis, this case appears to occur after an episode of acute necrotizing pancreatitis post-cholecystectomy. There is a lack of data about acute postoperative pancreatitis after LC although one cohort study reported that the incidence of pancreatitis after completed LC was 0.34% [12]. Acute pancreatitis after LC in the early postoperative period is typically attributed to the passage of a missed stone or biliary sludge along the ampulla of Vater. However, in rarer cases such as this, biliary stones or sludge were absent on repeat imaging. A similar case report by Kumar et al. presents a case of transient pancreatitis after LC without findings of stones or sludge on repeat imaging, stating that transient obstruction undetectable by conventional cholecystographic techniques may cause up to 75% cases of idiopathic pancreatitis and hypothesizes occult biliary disease as the cause [13,14].

Another potential cause of transient pancreatitis after LC may be attributed to Sphincter of Oddi dysfunction (SOD), which is a group of clinical pain syndromes caused by sphincter contraction abnormalities. The sphincter of Oddi is a fibromuscular band that encircles the major duodenal papilla and controls the flow of pancreatic and bilious secretions into the duodenum. SOD involves dysmotility or stenosis of the sphincter of Oddi, leading to reduced flow of these secretions [15]. Not only is SOD theorized to contribute to the risk of developing acute pancreatitis, SOD also increases the risk of post-ERCP pancreatitis [16]. While the prevalence of SOD is currently unknown, SOD is a known complication of cholecystectomies with ongoing biliary and pancreatic pain occurring in 10-20% of patients with prior cholecystectomies [17].

Whether this case is a result of a biliary pathology stemming from a pancreatic origin or vice versa is still unclear. However, the pseudocyst formation in this case appears to be a complication of acute pancreatitis rather than chronic pancreatitis. As the initial precipitating factor of pancreatitis in this case is unknown in addition to the limited number of similar cases reported in literature, further research into the pathophysiology of uncommon presentations of pancreatitis and subsequent pseudocyst formation is warranted.

Diagnosis of pancreatic pseudocysts is largely through imaging modalities. Laboratory evaluation is somewhat limited as elevated serum enzymes do not rule in the diagnosis of a pseudocyst; identification is based on a combination of clinical suspicion and imaging. Transabdominal ultrasound may be used as an initial study with a sensitivity ranging from 70-90%, though its accuracy is user-dependent. Contrast-enhanced CT is the modality of choice due to improved sensitivity from 82-100% and a specificity around 98% [1]. CT grants improved visualization of surrounding biliary structures, calcifications, and possible walled-off necrosis. However, the biggest limitation of the CT is the inability to distinguish between pseudocysts and neoplastic cystic lesions [1]. Another useful resource is the EUS, which provides closer and more detailed images, as well as allowing for diagnostic and therapeutic drainage of the pseudocyst. The MRI-Magnetic Resonance Cholangiopancreatography (MRCP) is the most accurate imaging modality for evaluating pancreatic duct anatomy, but is not as routinely used since the CT usually offers adequate information for diagnosis [1].

The gold standard for treating an uncomplicated pseudocyst is conservative management. Spontaneous resolution is common, especially for pseudocysts after acute pancreatitis. Management includes dietary modifications to a low-fat diet, along with analgesics and antiemetics, with follow-up interval imaging to monitor for pseudocyst enlargement or other complications. Complicated pseudocysts, such as those that arise from chronic pancreatitis, do not typically resolve spontaneously. Factors including location, number, and coexistence of other local complications affect the chances of spontaneous resolution. Invasive intervention is typically required for management of complicated pseudocysts, with any intervention being delayed about 6 weeks to allow the pseudocyst walls to mature, making them more amenable to manipulation or surgical drainage [1].

The three main categories of invasive management for pancreatic pseudocysts are percutaneous, endoscopic, and surgical drainage/excision [18]. Percutaneous drainage should be considered only in patients who are critically- ill and cannot tolerate other procedures, or in patients with infected or complicated pseudocysts, as this method of intervention does not require maturation of the cyst walls. Endoscopic drainage is becoming the preferred treatment modality, as it is less invasive, has lower complication rates, and carries higher long-term success rates. Endoscopic treatments also have the advantage of the ability to dilate pancreatic strictures encountered during the procedure, as well as the ability to place stents. In the past, surgical drainage was the modality of choice for pseudocyst drainage. However, due to the rise of endoscopic methods, surgical drainage is typically now reserved for malignant cysts, recurrent pseudocysts, or pseudocysts that are difficult to access with endoscopy. The complication rates of surgical drainage have been shown to be similar to endoscopic drainage rates, but have higher treatment success rates [18,19].

## Conclusions

In conclusion, this case illustrates an uncommon etiology of pancreatic pseudocyst formation following uncomplicated LC, without evidence of retained biliary obstruction, stones or other identifiable causes. Although pancreatic pseudocysts are typically associated with chronic pancreatitis or significant pancreatic ductal injury, this case demonstrates that they may also arise as a complication of acute pancreatitis post-cholecystectomy, even in the absence of common predisposing factors. As most pseudocysts resolve spontaneously, conservative management remains the standard of care; however, recurrent symptoms and complex presentations warrant careful consideration of endoscopic or surgical intervention. This case adds to the limited body of literature documenting post-cholecystectomy pancreatitis complicated by pseudocyst development. Its rare etiology underscores the importance of maintaining a high index of suspicion for pancreatic pathology in order to optimize patient outcomes in a timely manner, while also using individualized, multidisciplinary management strategies. Further studies are warranted to better characterize the mechanisms underlying post-cholecystectomy pseudocyst formation and to guide evidence-based management in these rare but clinically significant cases.
